# On the utility of immobilized phenylarsine oxide in the study of redox sensitive cardiac proteins

**DOI:** 10.1038/s41598-025-00665-4

**Published:** 2025-05-03

**Authors:** Asvi Arora Francois, Xiaoke Yin, Shinichi Oka, Junichi Sadoshima, Manuel Mayr, Philip Eaton

**Affiliations:** 1 William Harvey Research Institute, Queen Mary University of London, Charterhouse Square, London, EC1M 6BQ UK; 2 National Heart and Lung Institute, Imperial College London, London, SW7 2AZ UK; 3 Department of Cell Biology and Molecular Medicine, Rutgers New Jersey Medical School, Newark, NJ 07101 USA

**Keywords:** Heart, Redox, Thiol, Disulfide, Signalling, Phenylarsine oxide, Thioredoxin, Biochemistry, Analytical biochemistry, Proteomic analysis

## Abstract

Reactive protein cysteine thiols are critical to sensing and transducing oxidant signals, often by induction of disulfide bonds that alter their activity or interactions. Identifying such redox active proteins nowadays is mostly achieved using thiol redox proteomics with such datasets increasingly available. Subsequently, we are challenged with determining how changes in the redox state of a protein of interest alters its activity or interactions and how this affects physiology or disease progression including in vivo scenarios. Such studies necessitate the measurement of how the protein redox state changes with health or disease-related interventions, with it not always being practicable to resort back to resource-intensive proteomics to achieve this. In some proteins, oxidation to a disulfide state causes a non-reducing gel-shift, but this is mostly not the case and so other efficient approaches are required to index changes in redox state. Here we assessed the utility of immobilized, solid-phase phenylarsine oxide (PAO-Sepharose) as a tool for indexing the thiol redox state of candidate proteins in cardiac samples from in vivo interventions associated with oxidative stress. PAO-Sepharose, which binds proteins with proximal reduced thiol pairs but not when they form a disulfide, was also used to identify proteins that that are oxidised in isolated perfused mouse hearts exposed to hydrogen peroxide or diamide using proteomics. This together with complementary studies using a cardiac-specific FLAG-Thioredoxin-1C35S-HA transgenic ‘trap-mutant’ mouse model allowed identification of heart proteins susceptible to oxidant-induced disulfide bond formation using proteomics. Thus, two in vitro approaches identified putative cardiac thiol redox sensor proteins that were then assessed with in vivo follow-up studies for their susceptibility to oxidation during endotoxemia induced by lipopolysaccharide or type I diabetes induced by streptozotocin in mice. Of five proteins selected for further analysis by PAO-Sepharose binding, two, namely apoptotic protease activating factor 1 interacting protein (APIP) and γ-glutamylcyclotransferase (GGCT), displayed significantly lower affinity capture from hearts from lipopolysaccharide- or streptozotocin-treated mice, consistent with oxidation of their vicinal thiols. We conclude that PAO-Sepharose is an effective and accessible tool for identifying oxidant-sensitive protein thiols in both ex vivo and in vivo models of oxidative stress. As increasing numbers of thiol redox proteins are identified, PAO-Sepharose binding is an efficient method to determine if they change their oxidation state during interventions relevant to health and disease.

**Supplementary Information:**

The online version contains supplementary material available at 10.1038/s41598-025-00665-4.

## Introduction

Cells, including those of the heart, generate a diverse array of oxidant species during health and disease. Whilst there is evidence that these reactive species contribute to the pathogenesis of disease, it is now understood they can serve as signals that may enable homeostasis or adaptation to stress. Although oxidants are closely connected with atherosclerosis^1^, ischemia and reperfusion injury^2^, hypertension^3^, inflammation^4^, hypertrophy and progression to heart failure^5^, they also regulate myocardial contractile function^6^, and resistance to infarction^7^. Identifying proteins that sense changes in cellular redox status is an important step in defining how oxidant signals are integrated and contribute to cardiovascular regulation, disease progression or mechanisms that limit injury.

Oxidants can be sensed via their reaction with protein amino acids to introduce post-translational oxidative modifications, which can couple to changes in activity or interactions that contribute to cellular responses to these reactive species. Whilst many amino acids are susceptible to oxidative modification, the principal targets are thiol (-SH) side chains of cysteine residues^8^. Oxidation of cysteines is not indiscriminate because oxidants principally react with thiols with a low acid dissociation constant (p*K*_a_) that occur when deprotonation is stabilized by a vicinal acceptor side chain that occur on lysine, arginine, or histidine residues. These deprotonated cysteines exist in the ionized thiolate (S^−^) state that facilitates their heightened rates of reaction with oxidants that culminate in oxidative modifications. Indeed, there is evidence for sequence motifs that are susceptible to specific types of oxidations^9^. Disulfides, which can form between two adjacent cysteines within a protein as intradisulfides or between subunits as interdisulfides, are reduced back to the -SH state by the thioredoxin system - providing reversibility - a crucial feature of a regulatory system. Reversible formation of disulfide bonds can serve as a regulatory switch thereby allowing such proteins to sense their redox environment and couple this to a functional change (i.e., transduce) that mediates cellular homeostatic or maladaptive responses.

A variety of techniques are available for assessing the oxidation status of protein thiols, many of which are integrated with proteomics to provide a comprehensive overview of proteins undergoing redox state changes^10–14^. Multiple methods exist that allow the analysis of different oxidative modifications such as S-nitrosation^15–17^, S-sulfenation^11,18,19^, S-persulfidation^20,21^, and our interest here, namely proteins that form disulfides^22–24^. Indeed, comprehensive proteomics-derived lists of potentially cysteine redox regulated proteins are available, but the functional consequences and their potential impact on health and disease typically are not elucidated. Further exploration of a target of interest can be hampered by the fact that its redox state cannot readily be determined without resorting back to the oftentimes complex labelling and mass spectrometry used to initially identify the redox-modulated target. Such methods are resource intensive and time consuming and so may hinder follow-up studies, an issue partially addressed by the approach described here. Furthermore, candidate redox-regulated proteins are often identified using ex vivo model systems involving exposure to extracellular oxidants, whereas subsequent studies assessing the in vivo physiological or disease relevance require the redox state of the target to be routinely assessed in multiple, complex biological tissues. These tissues are often challenging to analyse using the initial mass spectrometry-based methods, which can be both resource-intensive and not always accessible or affordable for routine follow-up studies.

**Fig. 1:**
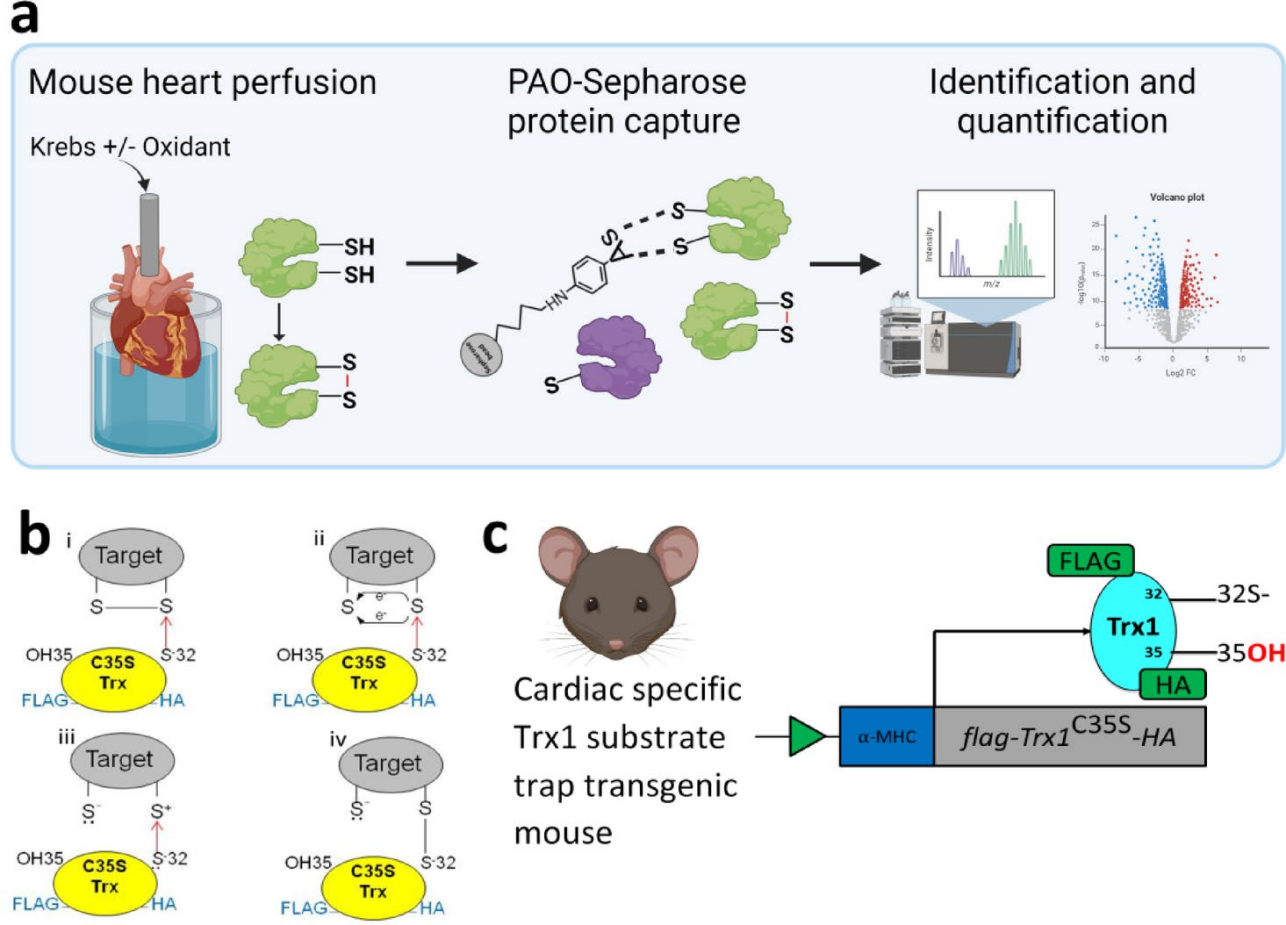
Proteomic strategies used to identify proteins with redox-sensing thiols. (**a**) Workflow of PAO-Sepharose capture of redox-sensitive proteins and their identification. (**b**) Cys35Ser Trx1 binds normally to the target and forms a disulfide bond (i-iii). The serine 35 residue however cannot reduce the disulfide bond like the wild type Trx with a cysteine at this site usually would (iv). The Cys35Ser mutant forms a stable protein complex with the oxidised target. (**c**) Summary of the FLAG-Trx1C35S-HA mouse that can ‘trap’ oxidised target proteins, with such proteins then being immune-enriched via the affinity tag before proteomic identification of associated proteins that represent those that form disulfides during oxidative interventions. Figure created with a licensed version of BioRender.com.

Although vast numbers of proteins undergo reversible thiol oxidations in a context dependent manner^25–30^, the availability of methods to routinely monitor their redox state in multiple, complex samples hampers the field. Trivalent arsenicals such as phenylarsine oxide (PAO), which complex with proximal pairs of thiols, represent a valuable tool in identifying such proteins, which have the potential to form disulfides when oxidants increase^22,31–34^. A key feature exploited in this study is PAO forms a stable diarsine ring with reduced vicinal thiols but not when they form a disulfide bond after an oxidative intervention^35^. Thus, cellular proteins that are captured by PAO-Sepharose, but not after an oxidative intervention, are likely to represent sensor proteins that form disulfides. Solid phase PAO-Sepharose was synthesized and used to capture proteins with vicinal thiols from isolated perfused mouse hearts after control aerobic perfusion or after chemical oxidative stress that induced disulfide bonds that decreased their affinity capture as quantified using mass spectrometry (Fig. 1a).

Thioredoxin (Trx) contains a pair of vicinal cysteines in its active site that are evolutionarily conserved^36^. This cysteine pair can exist in a reduced state (Trx-SH) or become oxidized to form an intradisulfide bond, with this cyclical redox process underlying its oxidoreductase activity. Oxidized targets undergo nucleophilic attack by the reduced Cys32 of Trx1, breaking the disulfide in the protein by a thiol-disulfide exchange reaction and causing a new disulfide complex to form as shown in Fig. 1b. This hetero-disulfide is short lived because the proximal Cys35 rapidly reduces this bond, generating a Cys32-Cys35 intradisulfide within Trx^37^, culminating in reduction of the target protein and oxidation of Trx1 which is then reduced by Trx reductase. In the present study, a transgenic mouse model was used that harbours a cardiomyocyte-specific mutation in Trx1 (Cys35Ser) downstream of a FLAG tag sequence (Fig. 1c)^38^. The Cys35Ser mutation results in incomplete Trx1 activity, where Cys32 initiates the disulfide exchange by forming an intermolecular bond with the cysteine in the target protein, but this bond cannot be resolved as it lacks the critical Cys35. As a result, a stable Trx1-substrate intermediate is formed, effectively ‘trapping’ substrates that originally contained a disulfide (Fig. 1b), which can then be enriched by immunocapture of the FLAG epitope.

Both the PAO-Sepharose and the Trx1 Cys35Ser-based experimental strategies provided a list of candidate cardiac proteins that likely form a disulfide in response to exogenous oxidants. However, the critical question as to whether any of these candidates are oxidized during scenarios of in vivo endogenously generated oxidative stress remained. Oxidation of some candidates of interest in hearts of mice exposed to lipopolysaccharide (LPS, endotoxemia model) or streptozotocin (STZ, type I diabetes model) was identified using the PAO-Sepharose protein capture approach, illustrating its utility in identifying oxidant-sensitive protein thiols in ex vivo and in vivo models of oxidative stress.

## Results

### The heart contains oxidant-sensing proteins

**Fig. 2:**
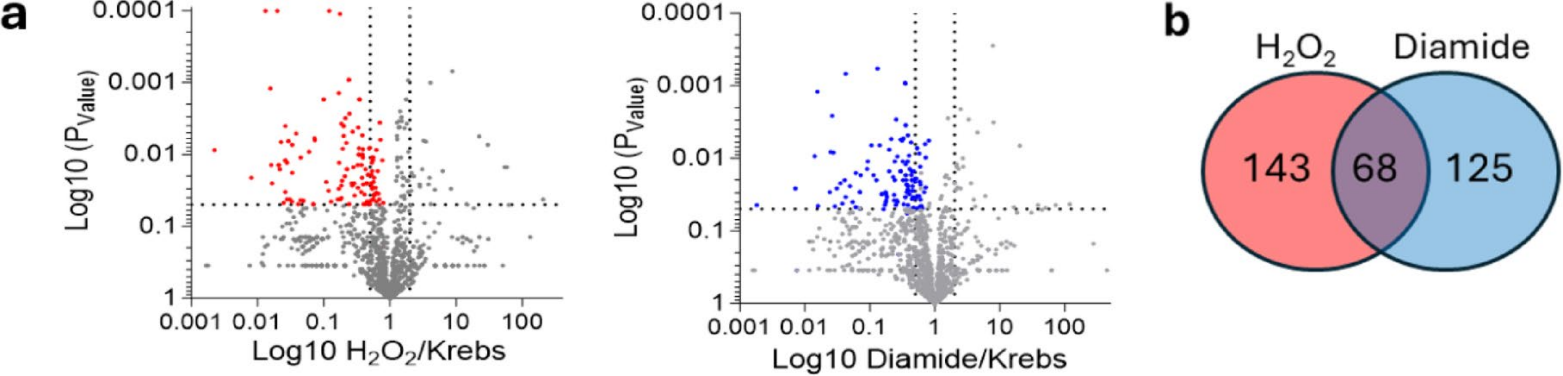
Proteins identified in Langendorff perfused mouse heart by LC-MS/MS after PAO-Sepharose capture. (**a**) Volcano plots of proteins captured by PAO-Sepharose from hearts isolated from C57BL/6J mice and subjected to Langendorff perfusion in the presence or absence of oxidants. Points in the top left quadrant represent proteins identified in the control group with > two-fold loss in the H_2_O_2_ (red) or diamide (blue) group which was statistically significant (*P* < 0.05). Statistical significance was calculated using an unpaired t-test. (**b**) Values in the Venn diagram indicate the number of proteins identified as statistically significantly oxidised by H_2_O_2_ (red) or diamide (blue) compared to vehicle control. The overlapping intersection indicates the number of proteins common to both oxidant interventions.

After retrograde perfusion of isolated hearts with or without the oxidant hydrogen peroxide or diamide, soluble fractions were prepared and applied to PAO-Sepharose. Proteins captured were identified and quantified using LC-MS/MS with spectral counting. A protein identified in the control (Krebs buffer alone) group which is subsequently lost (or captured less) after exposure to hydrogen peroxide or diamide is consistent with vicinal cysteine thiol oxidation to the disulfide state as shown in Fig. 1a. To highlight which of these proteins were reproducibly oxidized, volcano blots were generated by plotting the ratio of total spectra of an identified protein in an oxidant-treatment group vs. the control group against its P-Value (Fig. 2). The horizontal dashed lines show where *P* = 0.05 with points above the line showing statistical significance. Dots in the top left quadrant represent proteins that display a two-fold loss in capture after oxidant intervention which was statistically reproducible. The significant loss of a given protein the groups exposed to oxidants is consistent with it becoming oxidized to the disulfide state such that it no longer binds the PAO-Sepharose as shown schematically in Fig. 1a. Hydrogen peroxide or diamide significantly (*P* < 0.05) decreased the capture of 143 or 125 proteins respectively compared to control, representing targets that may mediate cardiac responses to oxidants and are presented in Supplementary Tables 1 and 2. Figure 2b presents a Venn diagram illustrating the number of proteins that undergo statistically significant oxidation following treatment with either H₂O₂ (red) or diamide (blue), compared to the vehicle control, using the PAO-Sepharose capture approach. The overlapping region highlights proteins common to both oxidant treatments. Dots in the top right quadrant represent proteins that unexpectedly exhibited increased PAO-Sepharose binding after exposure to oxidants. This may indicate the presence of vicinal cysteines that are normally concealed but become exposed following oxidant-induced modifications elsewhere in the protein. Additionally, vicinal cysteines could be masked by binding to partner proteins, with oxidant-induced alterations disrupting these interactions and enabling capture. Alternatively, oxidation may enhance protein-protein interactions, leading to the indirect detection of proteins bound to oxidized vicinal thiol-containing proteins.

**Fig. 3:**
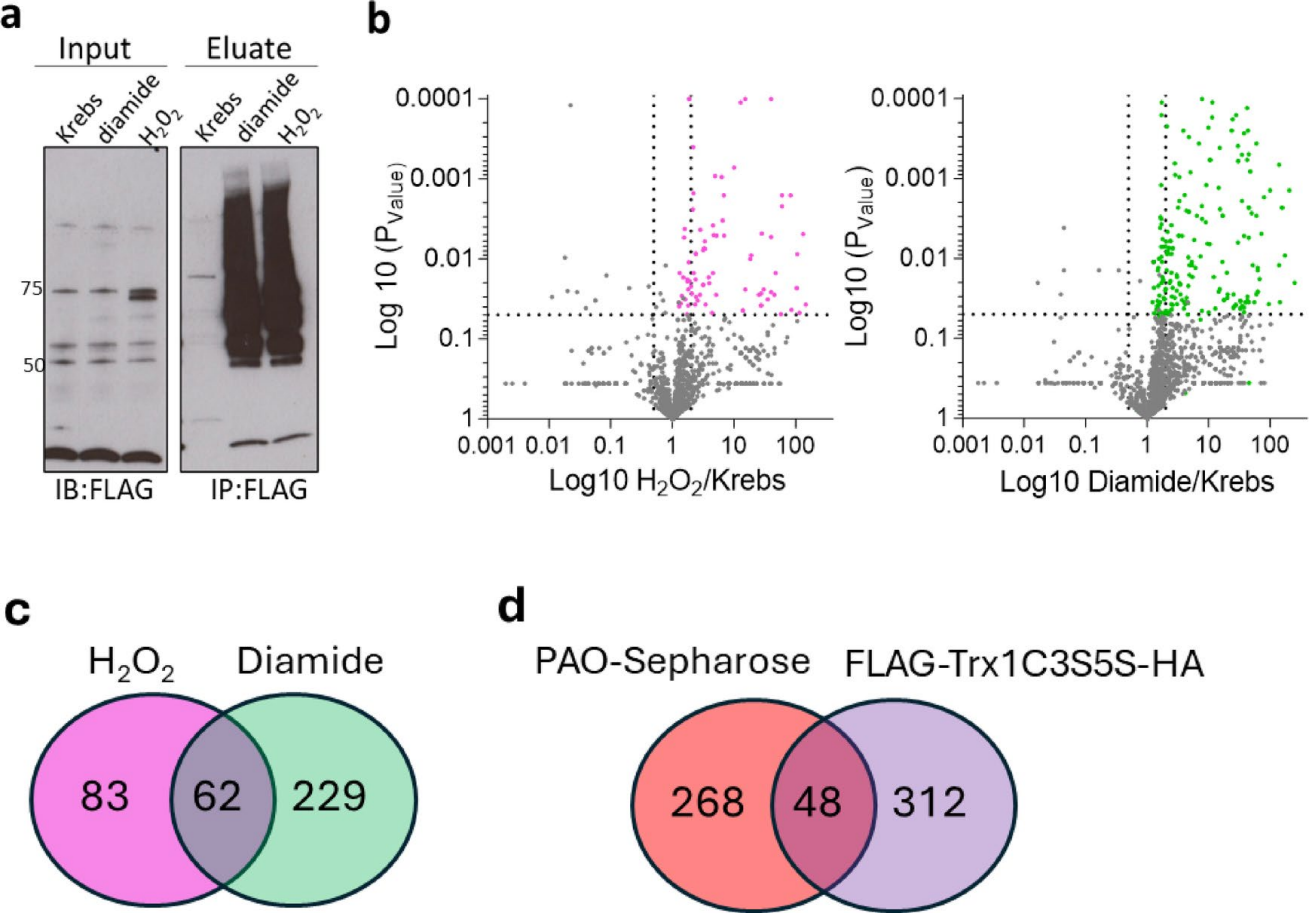
Interacting partners of FLAG-TrxC35S-HA after oxidant intervention. Hearts isolated from FLAG-TrxC35S-HA and subjected to Langendorff perfusion in the presence or absence of oxidants. (**a**) Stably trapped target proteins were isolated by FLAG immunoprecipitation and visualised by Western blotting. (**b**) Volcano plots of proteins identified by LC-MS/MS from the affinity capture shown in panel a. Points in the top right quadrant represent proteins identified in the control group with > two-fold increase in the H_2_O_2_ (pink) or diamide (green) group which was statistically significant (*P* < 0.05). (**c**) Values in the Venn diagram indicate the number of proteins identified as statistically significantly oxidised by H_2_O_2_ (pink) or diamide (green) compared to vehicle control. The overlapping intersection indicates the number of proteins common to both oxidant interventions. (**d**) A Venn diagram indicating the number of proteins identified as significantly oxidised by H_2_O_2_ or diamide using the PAO-Sepharose methodology (red) or the Trx1 Cys35Ser ‘trap-mutant’ (purple) analysis procedure. The overlapping intersection indicates the number of proteins identified that were common to both experimental approaches. Statistical significance was calculated using an unpaired t-test. Uncropped Western blots are included in the Supplementary information file.

In separate, complementary studies, hearts isolated from FLAG-Trx1C35S-HA mice were perfused with or without oxidants and soluble cardiac fractions were applied onto anti-FLAG affinity agarose to immunocapture FLAG-Trx1-substrate complexes. Eluates from hearts perfused with oxidants when probed with anti-FLAG antibody on western immunoblots produced additional bands when compared to Krebs buffer alone (Fig. 3a). Uncropped anti-FLAG Western blots are included in Supplementary Fig. 1. This demonstrates Trx1 forms stable complexes with proteins that had become oxidized through reactive cysteine moieties during oxidant intervention. Proteins captured in this manner were identified and quantified using LC-MS/MS with spectral counting. Dots in the top right quadrant of the volcano plots represent proteins that display a significant (*P* < 0.05) two-fold increase in capture after oxidant intervention (Fig. 3b). Such proteins are likely to be Trx1 disulfide-interacting partners that became ‘trapped’ after the oxidant intervention, as shown in Fig. 1b. Hydrogen peroxide or diamide significantly (*P* < 0.05) increased the capture of 83 or 229 proteins respectively compared to control, representing targets of Trx1 that may mediate cardiac responses to oxidants. These are presented in Supplementary Tables 3 and 4. Figure 3c shows a Venn diagram with the number of proteins that were significantly oxidized by H₂O₂ (pink) or diamide (green) compared to the vehicle control. The overlapping region indicates the number of proteins that are common to both oxidant treatments. Figure 3d shows a Venn diagram illustrating the number of proteins identified as significantly oxidized by H₂O₂ or diamide using either the PAO-Sepharose methodology (red) or the Trx1 Cys35Ser ‘trap-mutant’ approach (purple). The overlapping region represents the proteins commonly captured by each experimental approach.

## Well established cysteine redox-regulated proteins were identified by both methodologies

In an attempt to validate our findings, we searched for proteins with known reactive cysteines within the PAO-Sepharose data set. Tyrosine-protein phosphatase non-receptor type 11, a protein known to be regulated by reversible cysteine oxidation in its catalytic centre^39^, was identified in both oxidant groups (control vs. H₂O₂, *P* = 0.019; control vs. diamide, *P* = 0.017). Another example, Sterile Alpha Motif and Histidine-Aspartate Domain Containing Protein 1, which is also regulated through vicinal cysteine residues^29^, was identified in both oxidant groups (control vs. H₂O₂, *P* = 0.00035; control vs. diamide, *P* = 0.00068). These findings support the efficacy of the experimental approaches in identifying proteins with redox active cysteines that can form disulfide bonds when oxidant levels are increased.

Trx1 is a key component of the thioredoxin antioxidant reducing system, working in concert with other critical enzymes^40^. To validate the data obtained from the transgenic Trx1 hearts, we searched our proteomics lists for proteins that are known to interact with Trx1 through disulfide bond formation. Indeed, Peroxiredoxin 1 (control vs. H₂O₂, *P* = 0.00035) was identified, providing robust validation for the dataset. Additionally, several other Trx1 targets were also found. this included mitochondrial peptide methionine sulfoxide reductase, a known interacting partner of Trx1, which was identified in both groups from the FLAG-Trx1 screen (control vs. H₂O₂, *P* = 0.041; control vs. diamide, *P* = 0.00011)^41^. Furthermore, triosephosphate isomerase, a glycolytic enzyme that interacts with Trx1 during carbohydrate metabolism, was identified in both oxidant groups (control vs. H₂O₂, *P* = 0.016; control vs. diamide, *P* = 0.016)^42^. These findings provide additional confidence that proteomic analyses performed can truly identify proteins that that are susceptible to oxidation to the disulfide state during oxidative interventions. It is noteworthy that only peroxiredoxin 1 was identified as undergoing significant oxidation by H₂O₂ in the ‘trap-mutant’ study. However, the reason for the absence of other peroxiredoxins in this dataset, as well as in the thioredoxin ‘trap-mutant’ analysis, remains unclear. Furthermore, thioredoxins and peroxiredoxins did not show a decrease in capture on PAO-Sepharose in hearts exposed to oxidants. This was unexpected, as these proteins are known to undergo thiol oxidation under such conditions, and indeed we observe oxidation of many other protein cysteines that typically would be considered less sensitive to thiol oxidants. One possible explanation is that thioredoxin and peroxiredoxins cycle rapidly between reduced and oxidized states, preventing significant accumulation of either form under the conditions studied. Another consideration is that peroxiredoxins, upon exposure to hydrogen peroxide, may undergo hyperoxidation of their peroxidatic cysteine to sulfinic or sulfonic acid, which precludes disulfide formation. However, since such modifications would likely prevent PAO binding or engagement with the thioredoxin ‘trap-mutant,’ it remains unclear why there was no evidence for universal 2-Cys peroxiredoxin oxidation using both approaches.

## Modulation of redox sensitive proteins in pathophysiological models of oxidative stress

**Fig. 4:**
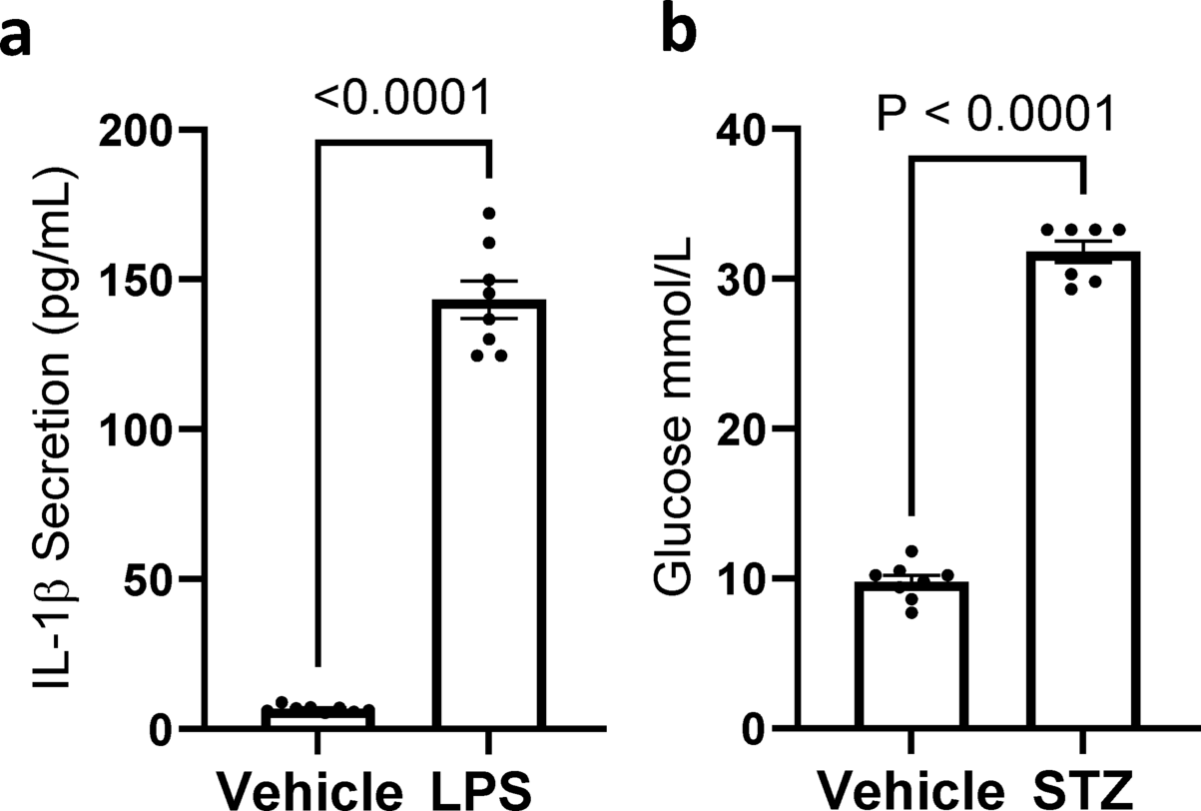
In vivo patho-physiological models of oxidative stress: (**a**) ELISA of IL-1β measured in plasma from mice administered LPS (endotoxemia model) or saline (*n* = 8). Statistical significance was calculated using an unpaired t-test. (**b**) Glucose measured in blood of mice administered STZ (type I diabetes model) or vehicle (*n* = 7–8). Statistical significance was calculated using a paired t-test. Data represents mean ± SEM.

The data shown in Figs. 2 and 3 were obtained from ex vivo models exposed to potentially supra-physiological concentrations of oxidants. While this approach facilitated a comprehensive analysis by enabling the detection of low-abundance redox-sensitive proteins and those forming transient disulfides, the applicability of PAO-Sepharose for analyzing samples from in vivo oxidative conditions remained unclear. Endotoxemia and Type I diabetes models in rodents are well-established to involve oxidative stress as a component of their aetiology^43–45^, and are also relevant to the human condition. To further evaluate PAO-Sepharose as a tool for identifying proteins oxidized in vivo under disease-relevant conditions, mice were subjected to LPS or STZ to induce more physiological or disease relevant oxidative events. Successful induction of endotoxemia was confirmed as plasma IL-1β significantly (P = < 0.0001) increased in mice exposed to LPS compared to vehicle controls (Fig. 4a). Similarly, Type I diabetes in mice was successfully induced by administration of STZ as corroborated by the significant (P = < 0.0001) increase in their non-fasting blood glucose compared to vehicle (Fig. 4b).

**Fig. 5:**
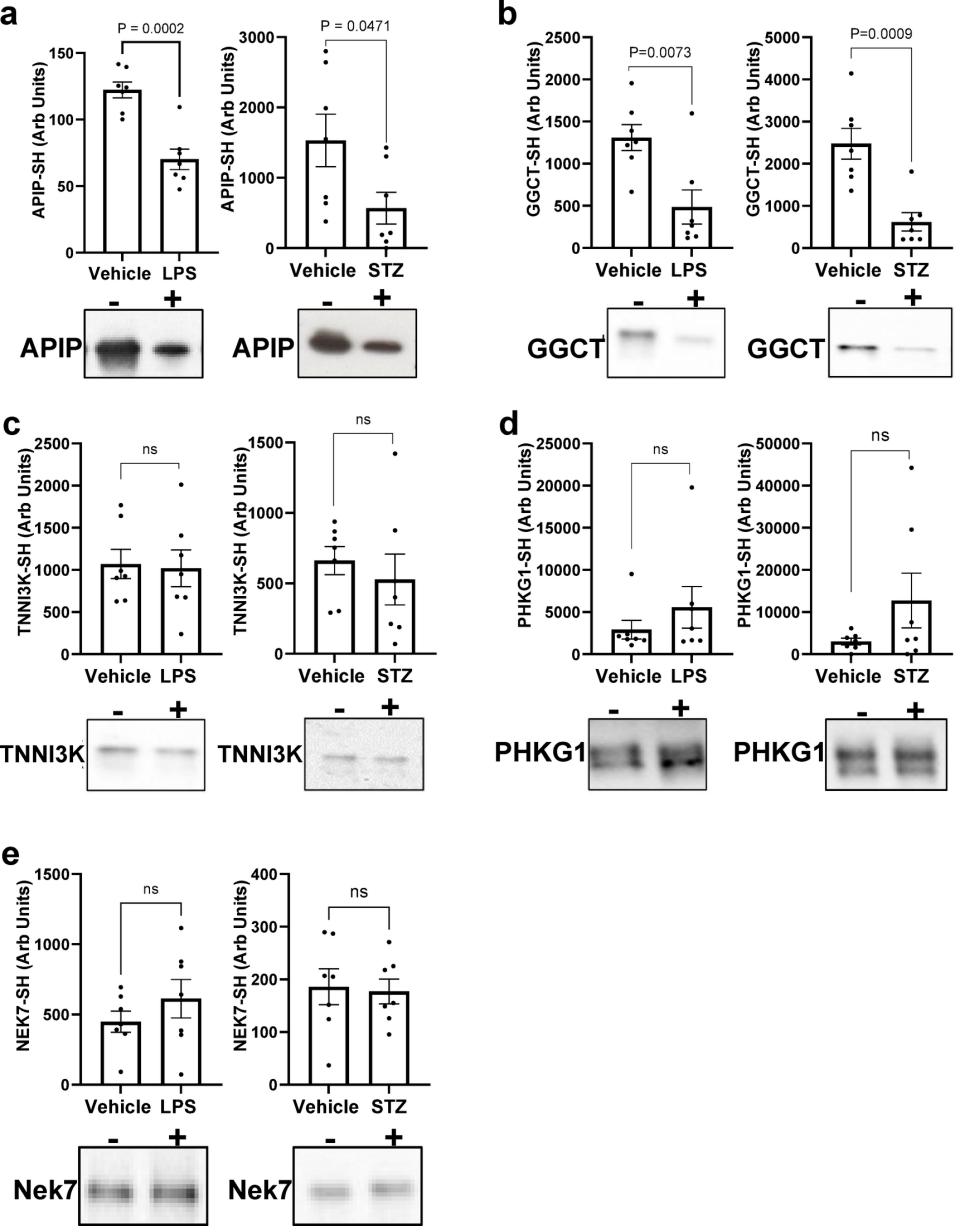
PAO-Sepharose capture of protein candidates from in vivo models of oxidative Stress. Proteins were assessed for changes to their redox state in hearts from mice exposed to LPS or STZ or vehicle alone. Eluates were probed with protein-specific antibodies to APIP (**a**), GGCT (**b**), TNN13K (**c**), PHKG1 (**d**) or Nek7 (**e**) and quantified using GelPro Analyser 3.1. A statistically significant loss (*p* < 0.05) of capture in the treatment vs. vehicle group was used as an index of thiol oxidation. Data represents mean ± SEM. Statistical significance was calculated using an unpaired t-test. Uncropped Western blots are included in the Supplementary information file.

Soluble fractions prepared from the hearts of these mice were applied to PAO-Sepharose columns and the capture of specific proteins of interest was quantified by immunoblotting using protein-specific antibodies. This approach was used to assess the redox state of selected proteins identified as susceptible to thiol oxidation in the proteomics studies, during in vivo exposure to LPS or induction of diabetes with STZ. Five proteins were selected from the proteomics data sets and their capture on PAO-Sepharose was evaluated as an index of their relative redox state under basal versus disease conditions. Our selection of targets was primarily guided by the availability of commercially available primary antibodies, rather than by their potential roles in the pathogenesis of endotoxemia or type I diabetes. Consequently, the protein targets analyzed via immunoblotting were chosen based on practical considerations, including their ability to be detect the protein of interest in mouse hearts with specificity, consistency and at the expected molecular weight. Apoptotic protease activating factor 1 interacting protein (APIP) was selected from the hydrogen peroxide group from perfused hearts and was chosen for further analysis in the in vivo models of oxidative stress. Immunoblot quantification revealed significantly reduced capture in fractions from mice treated with LPS (*P* = 0.0002) or STZ (*P* = 0.0471) compared to vehicle controls (Fig. 5a). This reduction in binding to PAO is consistent with APIP oxidation under these conditions. Similarly, γ-glutamylcyclotransferase (GGCT), was selected from the proteomics data set from perfused hearts. LPS (*P* = 0.0073) and STZ (*P* = 0.0009) treatment significantly attenuated GGCT capture compared to vehicle controls, indicating that it becomes more oxidised under these conditions (Fig. 5b). The proteins troponin I interacting kinase (TNN13K), phosphorylase b kinase gamma (PHKG1) and NIMA related kinase 7 (Nek7) were also assessed for changes in their redox state in the heart following LPS or STZ treatment, but no significant alterations were detected (Fig. 5c-e). Uncropped Western blots for each of these proteins assessed are included in the Supplementary Fig. 2.

## Discussion

This study presents two proteomic approaches aimed at gaining novel insights into redox-modulated proteins in the heart. In the first method, the arsenic compound PAO, which specifically binds to proteins with reactive cysteines, particularly vicinal thiols, was used. Thiols must be in their reduced state for complexes with PAO to form, but upon oxidation, reactive thiols form intra- or inter-disulfide bonds, preventing complexation with the arsenic moiety. This property was exploited by comparing the amounts of proteins capture under normal versus oxidising conditions, providing a list of potential thiol redox-modulated proteins in the heart. The second approach focused on identifying proteins in isolated hearts that increase their interactions with Trx1 upon exposure to oxidants provided in the buffer they were perfused with. By perfusing isolated hearts from transgenic mice with a ‘trap’ mutation^38^, we captured and identified potential cysteine oxidant sensor proteins in cardiac tissue.

**Fig. 6:**
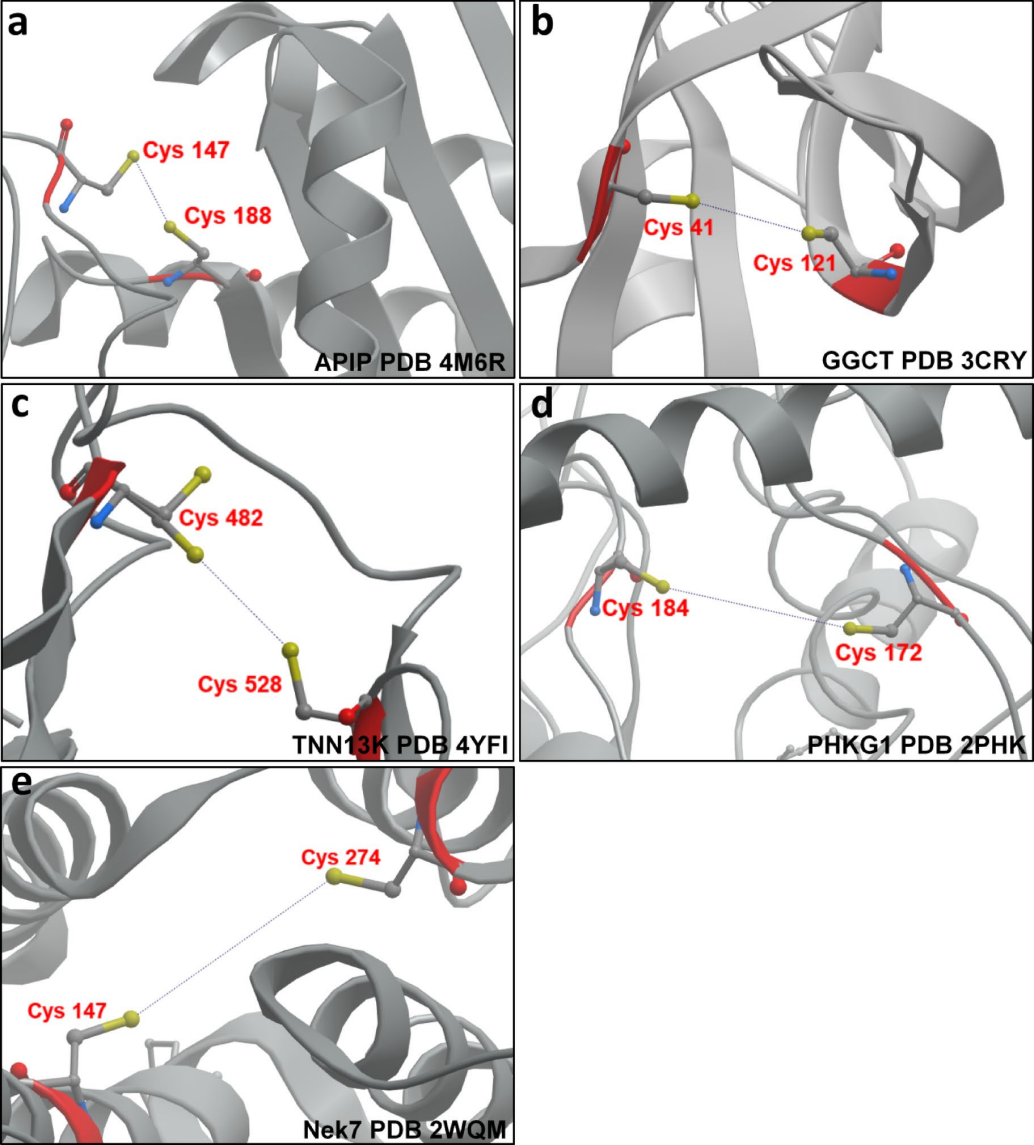
Potential intramolecular disulfide bonds in proteins captured by PAO-Sepharose as predicted by ReDisulphID.

Previous studies using immobilized PAO have provided extensive and detailed information on proteins containing vicinal thiols that are sensitive to reversible oxidation^22,31–34^. Much of this work has focused on the rat brain, but here, we have expanded these methods to identify novel thiol-switch proteins in the heart. The approach presented here is simple and accessible, though it has some limitations. PAO-Sepharose capture was performed on the soluble fraction of cardiac tissue, meaning redox-modulated proteins in the Triton-insoluble fraction were not analysed. Additionally, since no reducing or alkylating agents were used during the homogenisation process, there is a potential for auto-oxidation of thiols to occur during sample preparation, which could prevent their capture. As this affinity method does not specify the reactive cysteine residues that form complexes with PAO, additional approaches are necessary to pinpoint the sites of oxidative disulfide formation. After our analytical studies were completed, a new bioinformatics tool, ReDisulphID became available^46^. This platform predicts potential reversible redox-regulated disulfides across the entire mammalian Research Collaboratory for Structural Bioinformatics Protein Data Bank. We used ReDisulphID to evaluate the proteins studied here, namely APIP, GGCT, TNNI3K, PHKG1 and Nek7. Remarkably, the tool predicted that all these proteins contain vicinal cysteines, as shown in Fig. 6, which may be prone to reversible disulfide formation. Since these targets were selected for follow up before the advent of ReDisulphID, its prediction of possible vicinal, oxidation-susceptible cysteines in these very proteins reinforces confidence in the results from the two redox proteomics methods employed.

Investigators have employed approaches to catalogue Trx1 interacting partners using ‘substrate-trap’ models like the one described here^47,48^. Indeed, transgenic mouse models such as these coupled with MS approaches are powerful tools, but they require significant investment into generating and maintaining a transgenic mouse. An advantage of the ‘substrate-trap’ approach over the PAO-Sepharose method is that the interaction between Trx1 and its substrate occurs within the cell, maintaining the protein’s native environment, whereas PAO only binds reactive thiol moieties after cell disruption.

The rationale for using the two approaches was to identify redox-sensing candidates, but then the question remains whether these oxidations occur in vivo during times of oxidative stress. Physiologically, redox homeostasis is achieved by highly regulated cellular pathways with oxidative modifications such as inter- or intradisulfide bonds typically existing transiently because of the prevalent cellular reducing systems. During many pathologies however, this redox balance is perturbed leading to increased oxidation events and an altered cellular redox state. For instance, increased reactive oxygen and nitrogen species are generated from the aberrant activity of NADPH oxidase^49,50^, myeloperoxidases^51,52^, dysfunctional mitochondria^53,54^ and from a variety of immune cells^55^ during endotoxemia leading to redox imbalance^56^. Similarly, STZ models of Type 1 diabetes are well established to induce mitochondrial dysfunction^44,45,57^ and aberrant activity of xanthine oxidoreductase^45^, again leading to pro-oxidant alterations. Tissue obtained from mice subjected to LPS or STZ therefore allowed in vivo analysis of oxidative stress and assessment of candidate protein oxidation. Furthermore in vivo studies with PAO-Sepharose highlight its use in such scenarios of oxidative stress where the oxidative modification may be transient and under control of various cellular redox pathways.

APIP and GGCT were identified in the perfused heart dataset and found to be thiol redox modulated in hearts from mice subjected to endotoxemia or the STZ Type I diabetes model. APIP, a methylthioribulose 1-phosphate dehydratase, was identified in a chemoproteomic study where reactive thiols were quantified in various cell lines exposed to hydrogen peroxide^11^. This finding supports the idea that APIP may sense its redox environment via thiol groups that are prone to oxidation. Additionally, in a recent phosphoproteomic study, abnormal phosphorylation of APIP was observed in insulin-resistant men, consistent with a role for this protein in aberrant signalling contributing to the onset of diabetes^58^. This is particularly intriguing, as APIP was found to be redox modulated in the diabetes model presented here, hinting at a potential interplay between phosphorylation and oxidation in regulating the protein’s function. This provides confidence to the findings in this report and provides new mechanistic insight and avenues of future exploration.

GGCT thiol status was altered in hearts from mice exposed to LPS or STZ in this study, indicating this key enzyme to be sensitive to its redox environment in vivo. GGCT is a key enzyme involved in the synthesis of glutathione^59^, and clinically it is a biomarker for alcohol-induced liver disease^60^. Serum GGCT levels are also elevated in many cardiac pathologies including heart failure^61^, atherosclerosis^62^ and myocardial infarction^63^, though its precise role is not fully understood. While GGCT mRNA expression is upregulated during oxidative stress [71], it remains unclear whether its function is regulated by redox changes. Indeed, a key question that requires further investigation is whether the thiol status of the enzyme can influence its functional activity to impact physiology. Other proteins that were selected from the proteomics list, namely TNNI3K, Nek7 and PHKG1, did not show a significant alteration in their thiol status following the two in vivo interventions investigated. This is likely because these targets, along with many others, were initially identified in a robust model of chemical oxidative stress, where isolated perfused mouse hearts were directly exposed to hydrogen peroxide or diamide. In these models, supraphysiological levels of oxidants promote widespread oxidation, enabling the generation of a comprehensive list of oxidant-sensitive proteins. However, the oxidative stress associated with endotoxemia, or diabetes is expected to be more subtle, with different sources and molecular species of oxidants formed compared to the isolated heart studies. Different diseases likely produce varying amounts and types of oxidants at distinct locations, meaning only a subset of the targets identified in the exogenously oxidant-treated hearts are likely to be oxidized in these conditions.

Xiao et al. developed Oximouse, a comprehensive landscape of global cysteine oxidation in various tissues^10^. Although their study provides hugely valuable information and insights, such omics studies are costly and require specialist and complex data analysis which is not generically available. Targets identified using the PAO method described here have also been reported on the Oximouse portal, providing additional confidence to the data presented herein. Therefore, the PAO-method allows candidate targets already identified and reported in databases such as OxiMouse to be examined in a routine, time and cost-effective manner in biologically important scenarios.

In summary, we have described a relatively simple, quantitative, and accessible method using immobilized PAO and immunoblotting to monitor the thiol redox status of proteins of interest, including in in vivo samples for biomedically important conditions. Furthermore, the approach can be generically expanded to investigate reactive cysteine proteins identified from other proteomic studies in tissues and model systems of choice.

## Methods

### Animals

All procedures were performed in accordance with the Home Office Guidance on the Operation of the Animals (Scientific Procedures) Act 1986 in the United Kingdom and were approved by a Queen Mary University Animal Welfare and Ethical Review Body. Mice were group-housed when possible in animal care facilities under a 12-hour light: dark cycle at 23° ± 2 °C with water and standard diet *ad libitum*. All experiments were performed in accordance with the ARRIVE guidelines.

## Transgenic mice

Transgenic mice expressing FLAG-Trx1C35S-HA were generated as described previously^38^. Briefly, the cDNA encoding FLAG-Trx1C35S-HA was inserted downstream of the α-myosin heavy chain promoter in order to achieve cardiac-specific expression.

## Langendorff perfusion of isolated mouse hearts

12 week-old male C57BL/6J mice (Charles River, UK Limited) or transgenic mice expressing FLAG-Trx1C35S-HA (described above and in^38^) were euthanized by intraperitoneal injection of 6.6% sodium pentobarbitone (250 mg/kg) pre-mixed with heparin (500 USP units) (*n* = 4). Hearts were rapidly excised, immediately mounted onto Langendorff apparatus, and retrograde perfusion was established at a constant pressure of 80 mmHg with Krebs-Henseleit buffer (Krebs) (in mM: 118.5 NaCl, 25.0 NaHCO_3_, 4.75 KCl, 1.18 KH*2*PO_4_, 1.27 MgSO_4_, 11.0 D-glucose and 1.4 CaCl_2_) equilibrated with 95% O_2_ and 5% CO_2_ at 37 °C. Hearts were paced at 550 beats per minute. A fluid-filled balloon inserted into the left ventricle was used to monitor contractile function. For treatment with oxidants, hearts were stabilized for 20 min before switching to Krebs buffer with 0.1 mM hydrogen peroxide or 0.01 mM diamide for 10 min. At the end of the protocol, hearts were rapidly dismounted and frozen in liquid nitrogen until further analysis.

### Endotoxemia model

10 week-old, male C57BL/6J mice (Charles River, UK Limited) were administered LPS from *E.coli* (Cell signalling Technology, USA) at 7.5 mg/kg or saline interperitoneally for 3 h (*n* = 8). Mice were euthanized by intraperitoneal injection of sodium pentobarbitone as described above. Hearts were excised and snap frozen in liquid nitrogen and stored at -80 °C till use. Blood was collected in heparin-coated tubes from the thoracic cavity and centrifuged for 5 min at 5000 *g* to obtain plasma. IL-1β was quantified in plasma using an IL-1β mouse ELISA kit (ThermoFisher Scientific, UK).

### Type 1 diabetes model

STZ was prepared in citrate buffer pH 4.5 at 50 mg/mL on the day of use. 8 week-old male C57BL/6J mice (Charles River, UK Limited) were injected daily with STZ (50 mg/kg) or vehicle interperitoneally for 5 days (*n* = 7–8). Food was removed 4 h before injection. Non fasting glucose levels and body weights were determined at the beginning and end of the experiment. Whole blood was taken from the proximal ventral tail vein for glucose measurement using a glucometer (Accu-Chek II, Boehringer Mannheim, Canada). 14 days later, mice were euthanized by an intraperitoneal injection of sodium pentobarbitone (250 mg/kg) as above. Hearts were excised and snap frozen in liquid nitrogen and stored at -80 °C until further analysis.

### Cardiac soluble fraction preparation

Hearts were removed from storage at -80 °C and homogenized in ice cold homogenisation buffer 10% (w/v) comprising phosphate buffered saline (PBS) pH 7.4, 100 mM NaCl, 1% v/v Triton X-100 and an EDTA-free protease inhibitor tablet with a Ystral homogenizer. Homogenates were centrifuged at 10,000 *g* for 10 min at 4 °C to obtain a soluble fraction.

### PAO-sepharose preparation and affinity capture

4-Aminophenylarsenic oxide synthesis was commissioned to SynInnova (Canada), which was coupled to Sepharose as described previously^22^. Briefly, 32.8 mg of 4-Aminophenylarsenic oxide was dissolved in 10 mL of methanol, combined with 4 mL of a 50% suspension in isopropanol of NHS-activated Sepharose 4 Fast Flow (VWR, USA), and incubated while rotating at room temperature for 2 h. Remaining activated esters were blocked by the addition of 100 µL ethanolamine and an additional 1 h incubation at room temperature. The suspension was washed by centrifugation at 4000 *g*, three times with 10 mL of methanol and either kept at 4 °C for long term storage or washed in 10 mL of cold homogenisation buffer for protein capture. The concentration of arsine oxide groups in the PAO-Sepharose was determined to be 2.4–2.8 mmol/L of a 50% slurry of the PAO gel, as determined by measuring the decrease in available DTT thiols using the dithionitrobenzoate (DTNB) assay^64^. Briefly, DTT was incubated with the PAO slurry for 10 min. Unbound DTT was then measured in the supernatant after removal of PAO-Sepharose by centrifugation. 1 mL of soluble cardiac fraction was applied onto 400 µL PAO-Sepharose (50% slurry in homogenisation buffer) for 90 min at 4 °C with rotation. After incubation, protein-PAO complexes were washed 5 times in wash buffer (PBS pH 7.4, 0.1% v/v Tween-20) by centrifuging at 4000 *g* for 5 min. Proteins were eluted directly in 500 µL 2X Laemmli sample buffer with boiling for 5 min.

### FLAG immunoprecipitation

1 mL of soluble cardiac fraction was applied onto 200 µL of anti-FLAG M2 affinity agarose (50% slurry in homogenisation buffer) (Sigma, USA) for 2 h at 4 °C with rotation. The gel was washed five times in wash buffer (PBS pH 7.4, 0.1% v/v Tween-20) by centrifuging at 4000 *g* for 5 min. Proteins were eluted directly in 500 µL 2X Laemmli sample buffer containing 100 mM maleimide with boiling for 5 min.

### Protein preparation for LC-MS/MS

Proteins were separated on 4–20% mini-protein TGX precast gels (Bio-Rad, USA) according to the manufacturer’s instructions and visualized by Silver Staining (Thermo Fisher Scientific, UK). Each lane was cut into 20 equally sized gel bands. Each gel band was diced into small pieces, reduced by DTT, alkylated by iodoacetamide and digested on a ProGest Protein Digestion Station (Genomic Solutions) using trypsin at 37 C overnight.

### LC-MS/MS

Digested peptides were separated by a nanoflow HPLC (U3000, Thermo Fisher Scientific) on a C18 reversed phase column (PepMap100, C18, 75 μm x 25 cm, Thermo Fisher Scientific) and analysed by LTQ Orbitrap XL mass spectrometer (Thermo Fisher Scientific). The HPLC flow rate was 300nL/min and the following gradient was used: 0–10 min, 4-10% B; 10–85 min, 10-30% B; 85–90 min, 30-40% B; 90–100 min, 100% B; 100–120 min 4% B; where A = 0.1% formic acid in LC-MS grade H2O and B = 80% acetonitrile, 0.1% formic acid in LC-MS grade H2O. For the MS method, full MS scan was acquired using Orbitrap at resolution 60,000, m/z range 350–1600. Six most abundant peaks from each full MS scan were selected for MS/MS using collisional induced dissociation and acquired using linear ion trap. Lock mass ion of m/z = 445.12003 was used. Dynamic exclusion was enabled: when the same precursor ion was fragmented 2 times within 30 s, it will be excluded for 120 s.

### Database searching

All RAW files were analyzed using Mascot (Matrix Science, London, UK; version 2.3.01). Mascot was set up to search the MOUSEsp201502 database (selected for Mus musculus, UniProt/Swiss-Prot version 2015_02, 16699 protein entries) assuming the digestion enzyme trypsin. Mascot was searched with a fragment ion mass tolerance of 0.80 Da and a parent ion tolerance of 10.0 PPM. Carbamidomethyl of cysteine was specified in Mascot as a fixed modification. Oxidation of methionine was specified in Mascot as a variable modification.

### Criteria for protein identification

Scaffold (version Scaffold_4.8.6, Proteome Software Inc., Portland, USA) was used to validate MS/MS based peptide and protein identifications. Peptide identifications were accepted if they could be established at greater than 90.0% probability by the Peptide Prophet al.gorithm^65^ with Scaffold delta-mass correction. Protein identifications were accepted if they could be established at greater than 99.0% probability and contained at least 1 identified peptide. Protein probabilities were assigned by the Protein Prophet al.gorithm^66^. Proteins that contained similar peptides and could not be differentiated based on MS/MS analysis alone were grouped to satisfy the principles of parsimony. Proteins sharing significant peptide evidence were grouped into clusters.

### Western blot analysis

Proteins were separated on 4–20% mini-protein TGX precast gels as described above and transferred on to PVDF membrane (Bio-Rad, USA). Immunoblots were probed with primary antibodies (diluted to 1:1000) to FLAG (14793) (Cell Signalling Technology, USA), APIP (ab154258), GGCT (ab198503), Nek7 (ab133514), TNNI3K (ab136954) or PHKG1 (ab194112) (Abcam, UK). Horseradish peroxidase–linked rabbit or mouse secondary antibodies (1:2000) (Cell Signalling Technology, USA) and Enhanced chemiluminescence Western Blotting Detection Reagent (GE Healthcare, USA) were used. Unaltered, original digitized immunoblots were quantitatively analysed using GelPro Analyser 3.1.

### Statistics

Results are presented as mean ± SEM unless stated otherwise. Differences between groups were assessed by unpaired t-tests using Scaffold (version Scaffold_4.8.6, Proteome Software Inc., Portland, OR) or GraphPad Prism.

## Electronic supplementary material

Below is the link to the electronic supplementary material.

Supplementary Material 1

Supplementary Material 2

Supplementary Material 3

Supplementary Material 4

Supplementary Material 5

## Data Availability

All data generated or analysed during this study are included in this article or Supplementary Figures 1-2 and Supplementary Tables 1-4.
