# Supplementary material for: On the utility of immobilized phenylarsine oxide in the study of redox sensitive cardiac proteins
Source: Sci Rep. 2025 May 3;15:15554. doi: 10.1038/s41598-025-00665-4 (PMC12049532; doi:10.1038/s41598-025-00665-4)
Supplement: Supplementary file 5 — Supplementary Material 5 [file 41598_2025_665_MOESM5_ESM.docx]

**Supplementary Information**

Krebs

diamide

H_2_0_2_

Krebs

diamide

H_2_0_2_

Krebs

Krebs

Input

Eluate


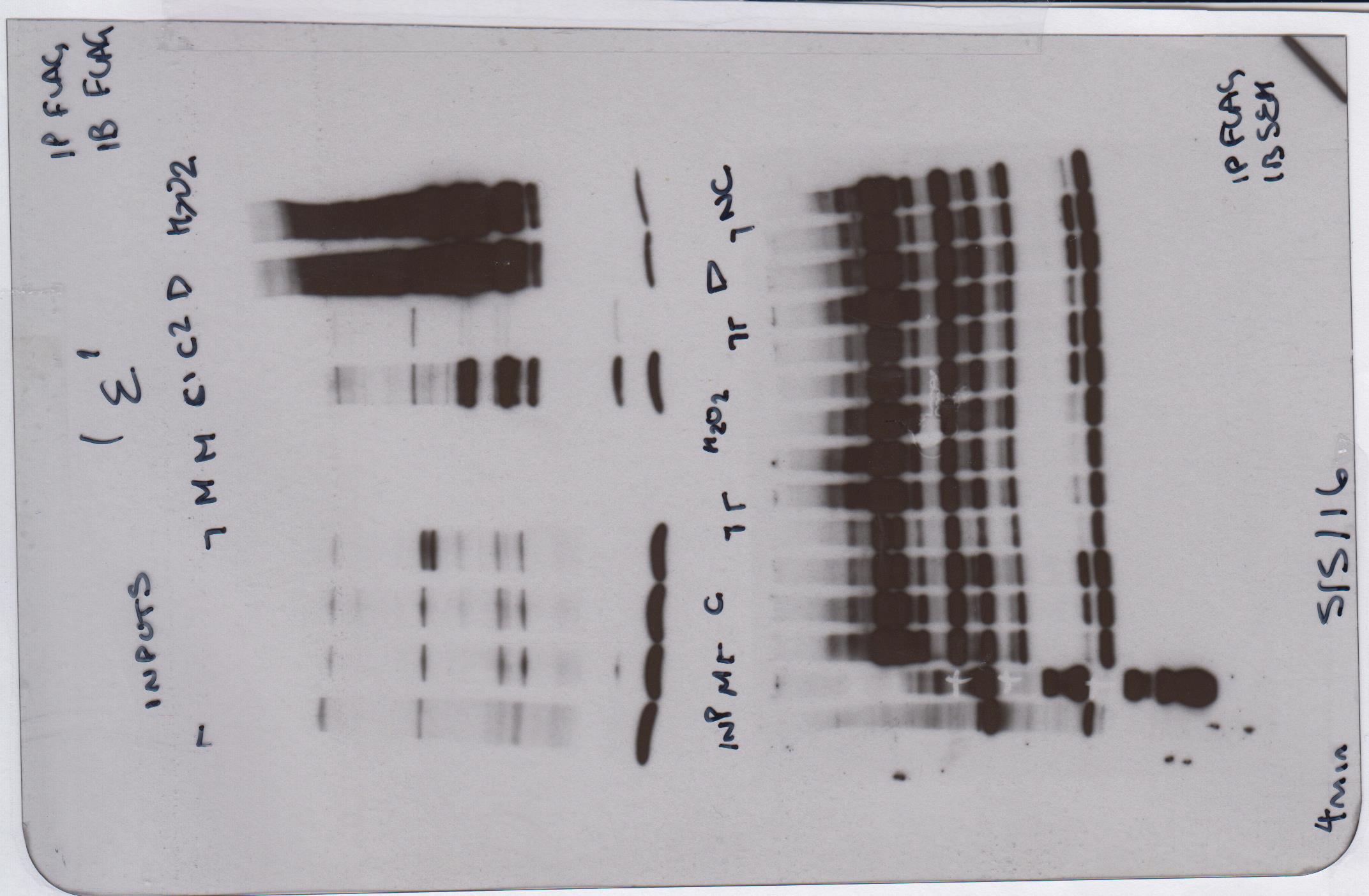


50

37

100

75

25

20

150

250

15

10

**a**

**IP:FLAG**

**IB:FLAG**

**Supplementary Figure 1: Uncropped Western blot image shown in Figure 3a.** Hearts isolated from FLAG-TrxC35S-HA and subjected to Langendorff perfusion in the presence or absence of oxidants. (a) Stably trapped target proteins were isolated by FLAG immunoprecipitation and visualised by Western blotting.

**a**

25

20


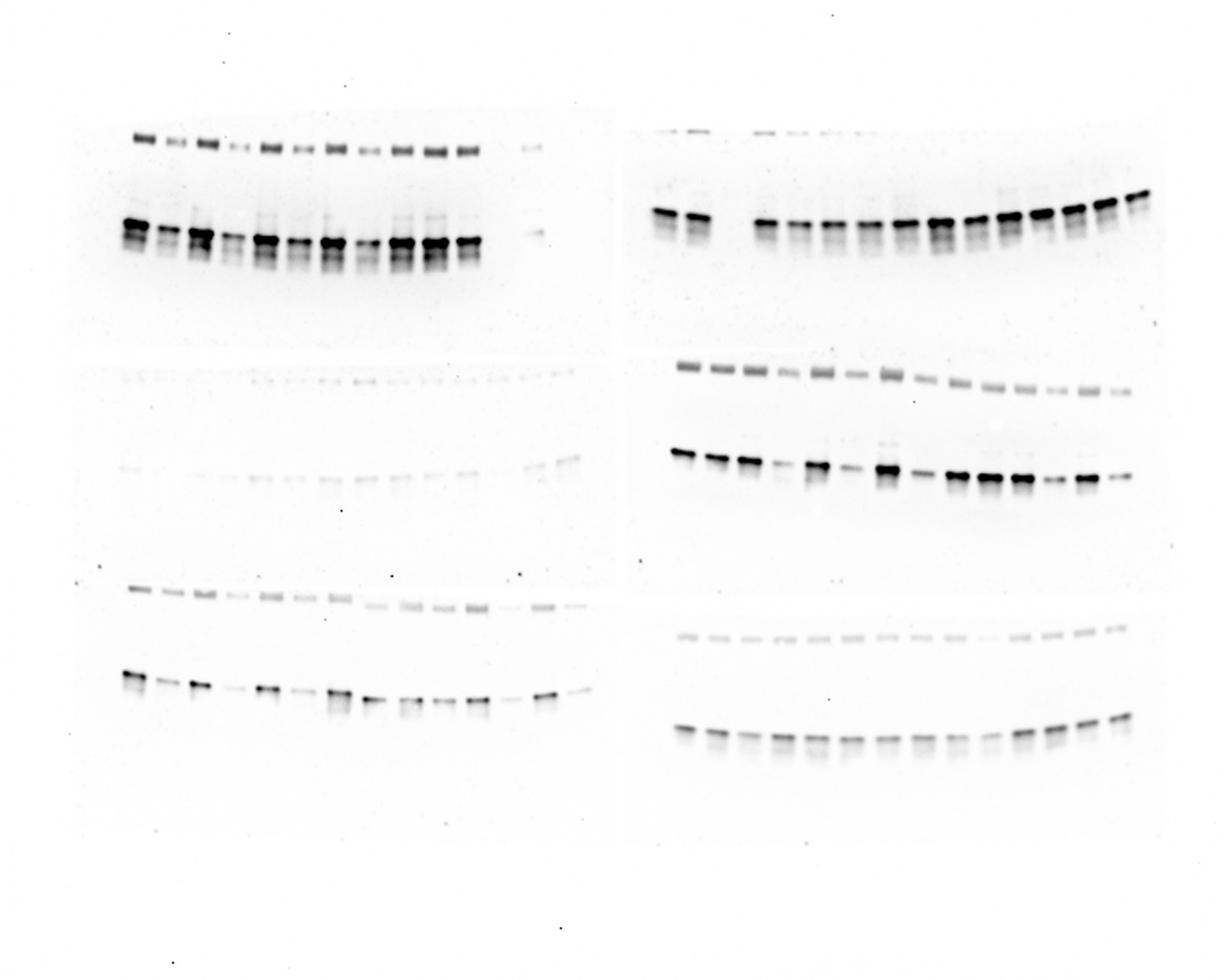

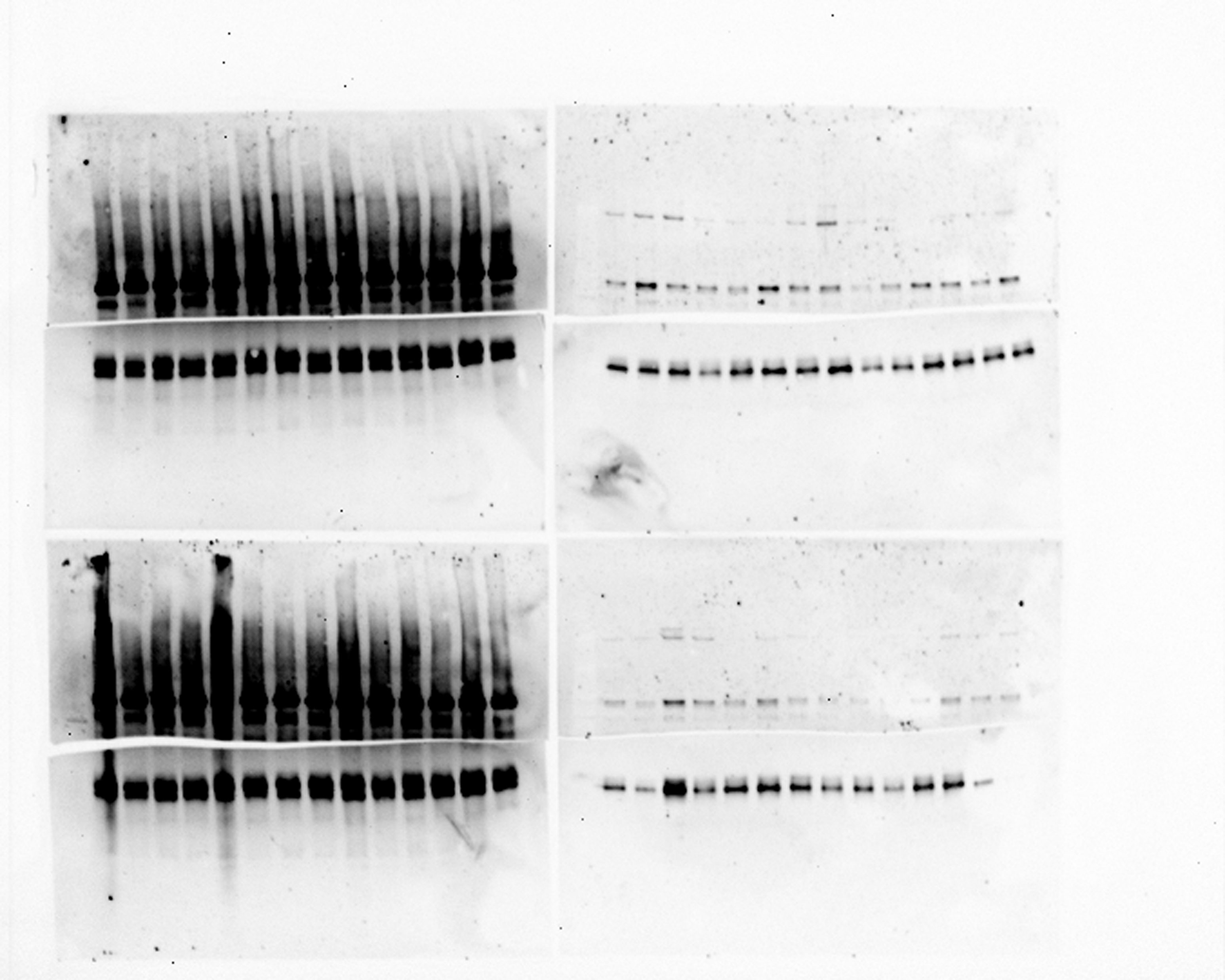

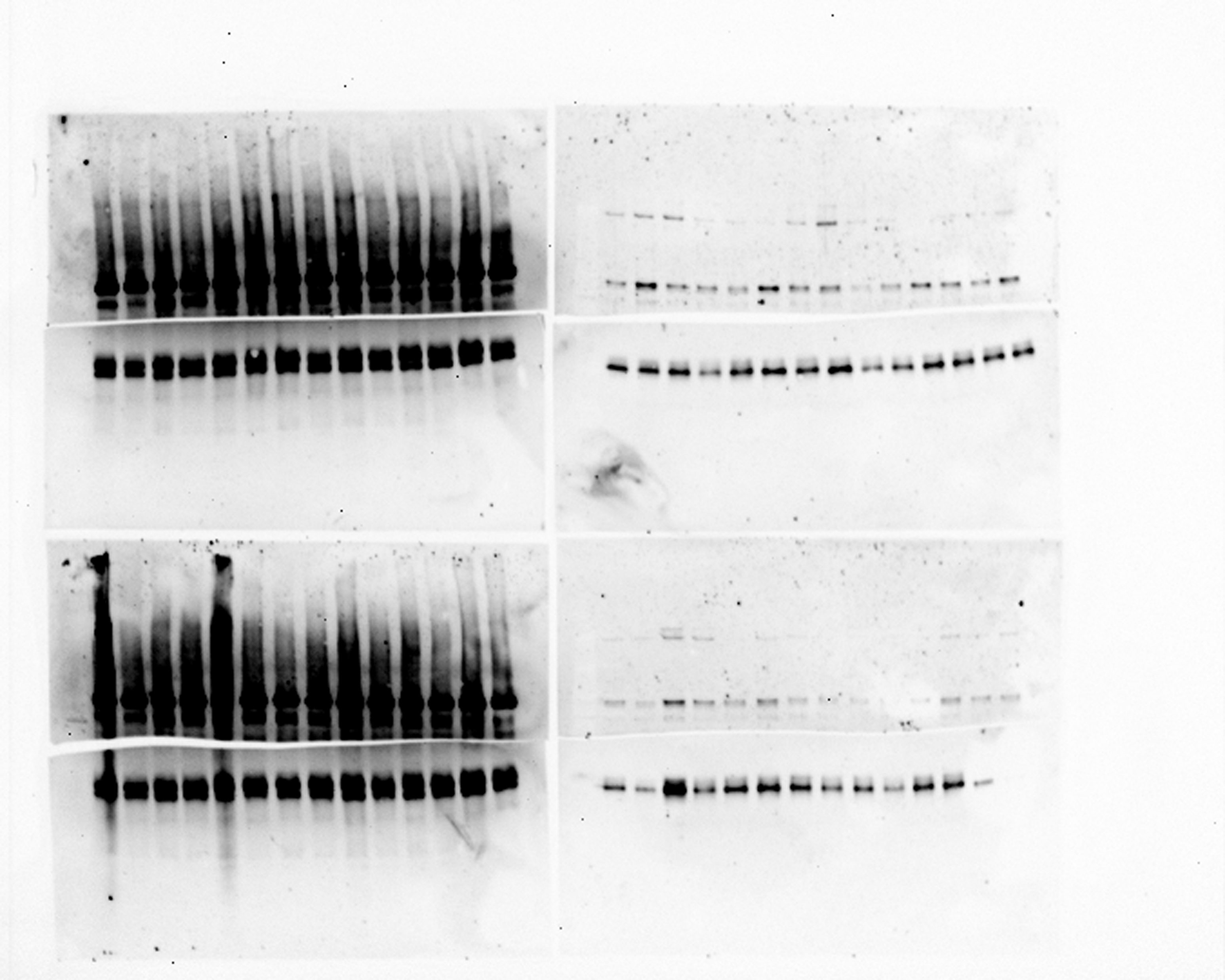


100

75


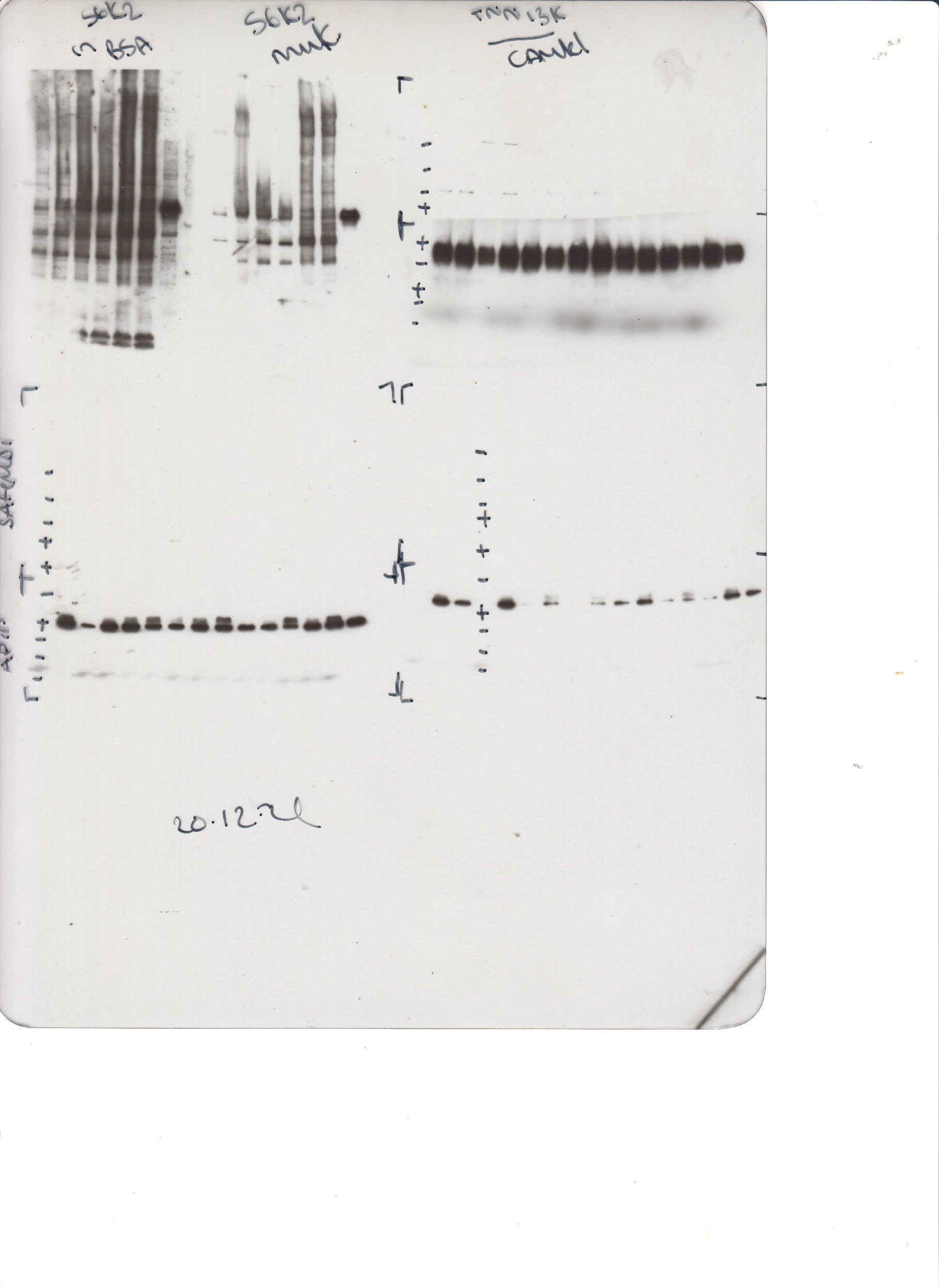


**c**

**+**

**-**

**+**

**+**

**-**

**-**

**-**

**-**

**-**

**-**

**+**

**+**

**+**

**+**

**STZ**

**+**

**-**

**+**

**+**

**-**

**-**

**-**

**-**

**-**

**-**

**+**

**+**

**+**

**+**

**STZ**


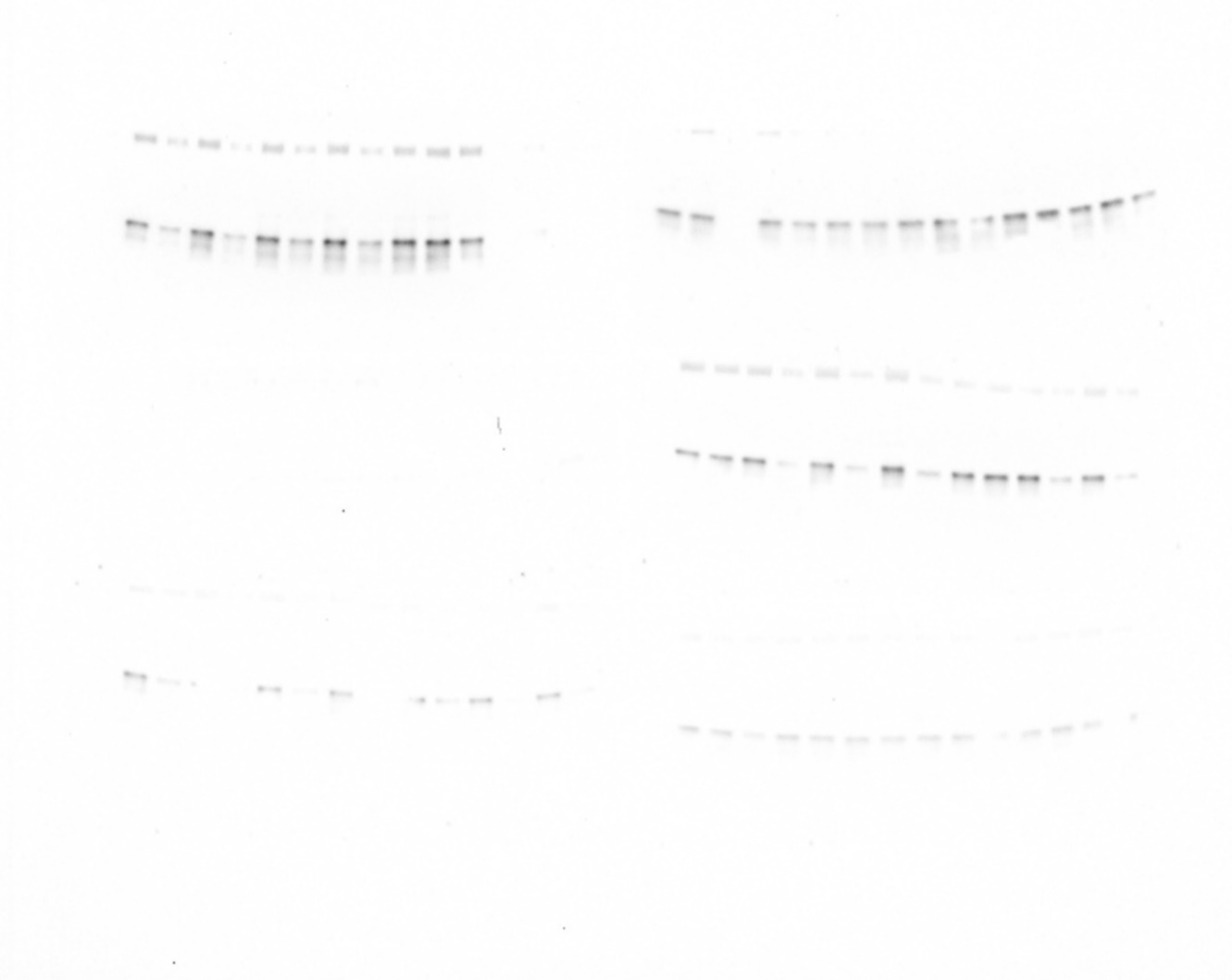


25

20

50

37

**b**

**+**

**-**

**+**

**+**

**-**

**-**

**-**

**-**

**-**

**-**

**+**

**+**

**+**

**+**

**LPS**

**+**

**-**

**+**

**+**

**-**

**-**

**-**

**-**

**-**

**-**

**+**

**+**

**+**

**+**

**LPS**


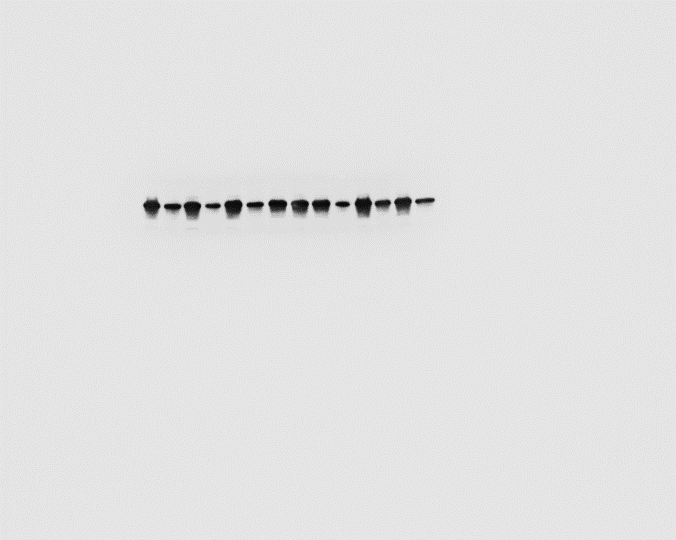


**+**

**-**

**+**

**+**

**-**

**-**

**-**

**-**

**-**

**-**

**+**

**+**

**+**

**+**

**LPS**

**+**

**-**

**+**

**+**

**-**

**-**

**-**

**-**

**-**

**-**

**+**

**+**

**+**

**+**

37

25

50

100

75

150

250

20

15

10

37

25

50

100

75

150

250

20

15

10

15

10

25

20

50

37

15

10

**STZ**

**d**


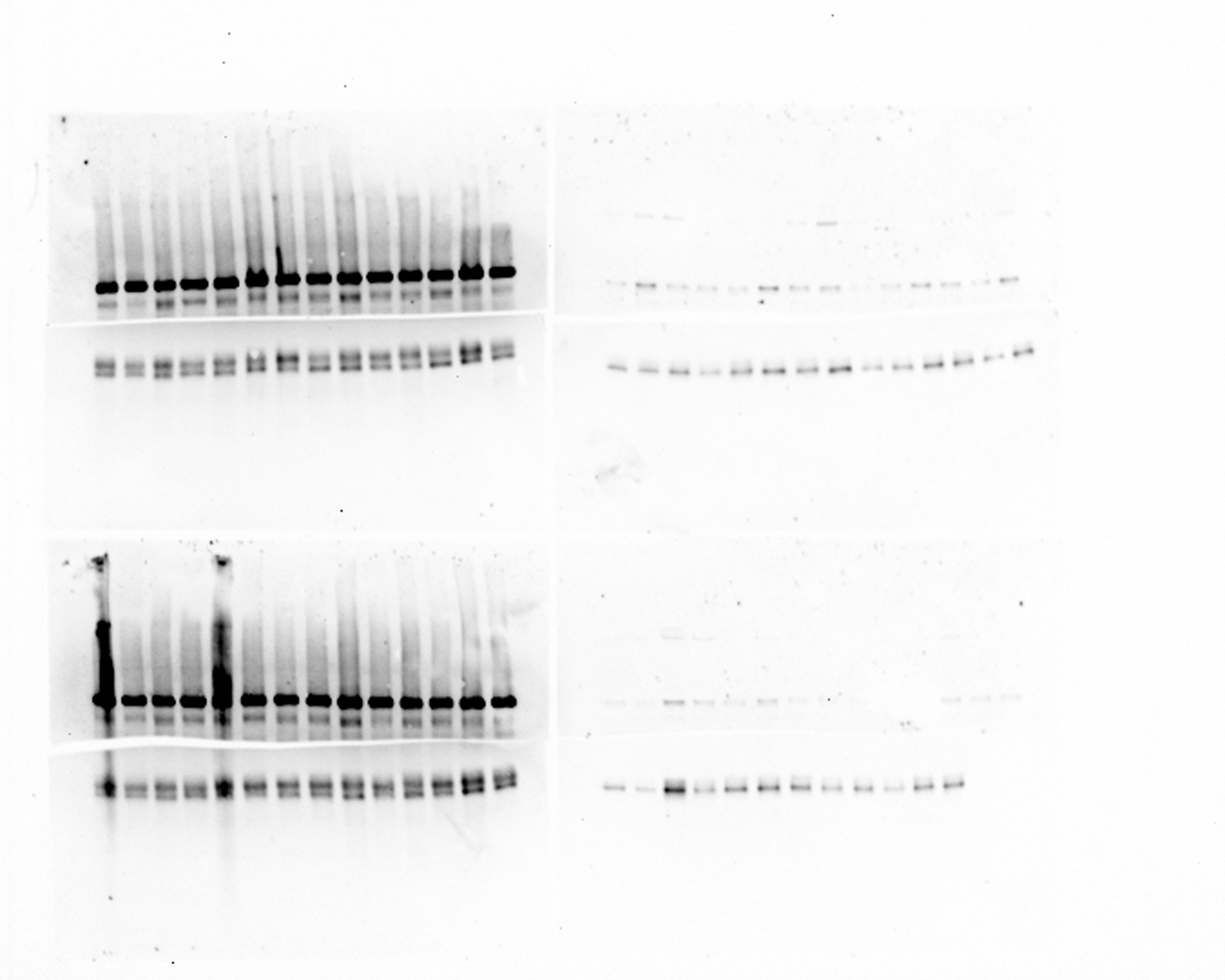


50

37


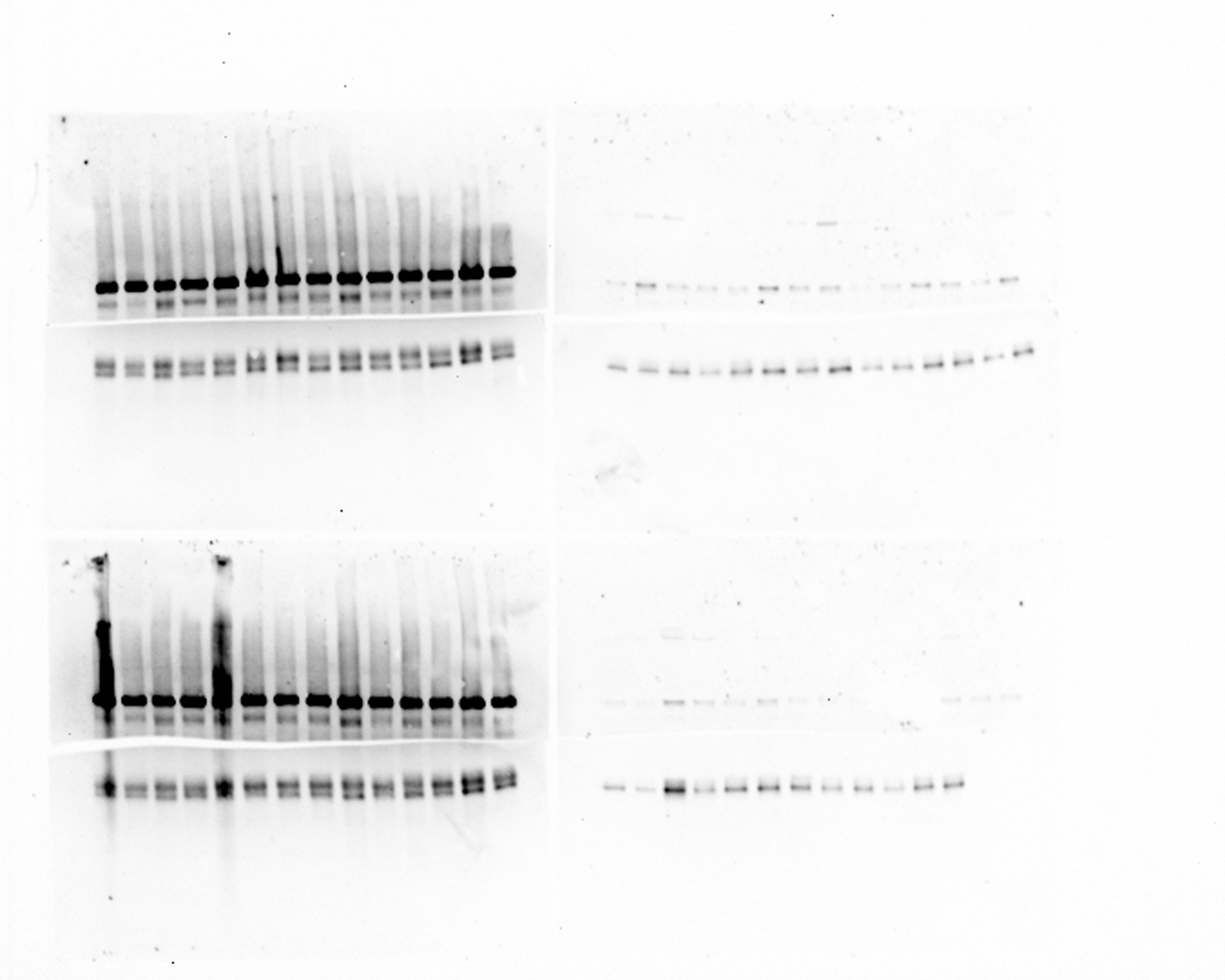

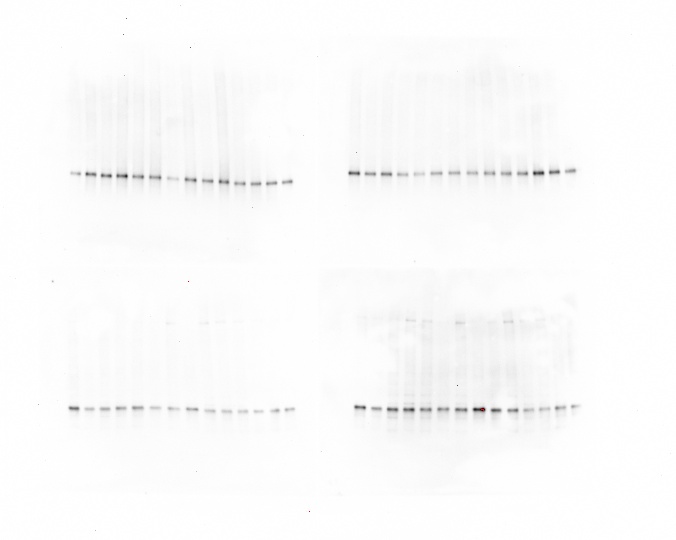

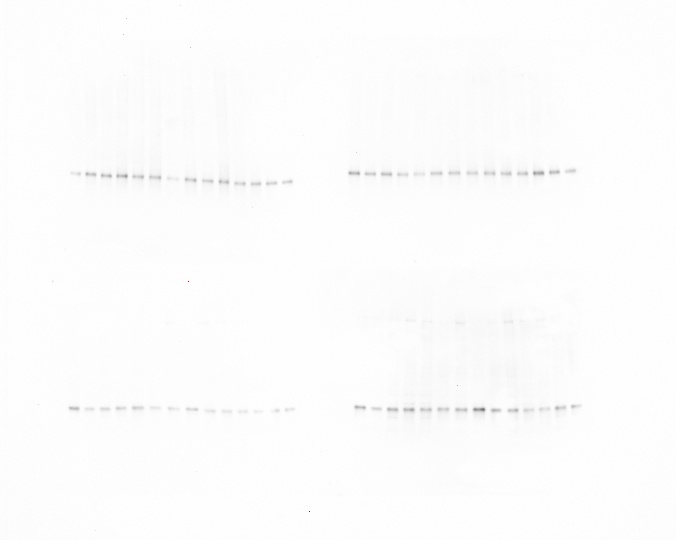


**LPS**

**e**

**+**

**-**

**+**

**+**

**-**

**-**

**-**

**-**

**-**

**-**

**+**

**+**

**+**

**+**

**+**

**-**

**+**

**+**

**-**

**-**

**-**

**-**

**-**

**-**

**+**

**+**

**+**

**+**

**+**

**-**

**+**

**+**

**-**

**-**

**-**

**-**

**-**

**-**

**+**

**+**

**+**

**+**

**+**

**-**

**+**

**+**

**-**

**-**

**-**

**-**

**-**

**-**

**+**

**+**

**+**

**+**

**LPS**

75

25

20

15

50

37

75

25

20

15

37

25

50

100

75

150

250

20

15

10

37

25

50

100

75

150

250

20

15

10

**STZ**

**STZ**

150

250

100

75

150

250

**Supplementary Figure** 2: **Uncropped Western blot images shown in Figure 5. PAO-Sepharose capture of protein candidates from *in vivo* models of oxidative Stress.**. Eluates were probed with protein-specific antibodies to APIP (a), GGCT (b), TNN13K (c), PHKG1 (d) or Nek7 (e) and quantified using GelPro Analyser 3.1.Arrows indicate the band of interest where necessary. Some membranes were cut prior to primary antibody incubation, and this is indicated by a dashed line.
